# Primary brainstem lymphoma: clinical characteristics, treatment methods, and prognostic factors of 56 patients

**DOI:** 10.1093/oncolo/oyaf105

**Published:** 2026-02-25

**Authors:** Wenhui Guo, Ling Duan, Shuo Yin, Jiefei Han, Hanyun Zhao, Shoubo Yang, Gehong Dong, Wenbin Li, Feng Chen

**Affiliations:** Department of Neuro-oncology, Cancer Center, Beijing Tiantan Hospital, Capital Medical University, Beijing, China; Department of Neuro-oncology, Cancer Center, Beijing Tiantan Hospital, Capital Medical University, Beijing, China; Department of Neuro-oncology, Cancer Center, Beijing Tiantan Hospital, Capital Medical University, Beijing, China; Department of Neuro-oncology, Cancer Center, Beijing Tiantan Hospital, Capital Medical University, Beijing, China; Department of Neuro-oncology, Cancer Center, Beijing Tiantan Hospital, Capital Medical University, Beijing, China; Department of Neuro-oncology, Cancer Center, Beijing Tiantan Hospital, Capital Medical University, Beijing, China; Department of Pathology, Beijing Tiantan Hospital, Capital Medical University, Beijing, China; Department of Neuro-oncology, Cancer Center, Beijing Tiantan Hospital, Capital Medical University, Beijing, China; Department of Neuro-oncology, Cancer Center, Beijing Tiantan Hospital, Capital Medical University, Beijing, China

**Keywords:** brainstem, lymphoma, chemotherapy, radiotherapy, survival, prognosis

## Abstract

**Background:**

Primary brainstem lymphoma (PBSL) is rare and generally has a poor prognosis. This study aimed to analyze the clinical, therapeutic, and prognostic factors in a single-center retrospective cohort.

**Methods:**

Fifty-six patients diagnosed with PBSL from January 2018 to December 2022 were retrospectively enrolled. Data on the clinical course of disease, pathological parameters, neuroradiological imaging, laboratory results, and survival data were collected and analyzed.

**Results:**

The median age at diagnosis was 56 years (range: 10-77), and 23 (41.1%) patients had a Karnofsky Performance Score ≤60. The mid-brain was the most commonly involved brainstem region (35.7%). The histological type of all patients was diffuse large B-cell lymphoma. The first-line treatment was high-dose methotrexate (HD-MTX)-based chemotherapy, with an overall response rate (ORR) of 70.5%, of which 62.1% achieved complete response. For relapsed or refractory disease, radiotherapy was the most common salvage therapy and significantly improved overall survival (OS). With a median follow-up of 56 months, the median OS for the entire cohort was 30 months (95% CI 18-42 months). In both univariate and multivariate analysis, an age of ≥60 years at diagnosis was a significant prognostic factor for poor OS (hazard ratio [HR] 3.086, *P* = .003, 95% CI 1.467-6.492) and progression-free survival (HR 2.309, *P* = .030, 95% CI 1.087-4.905).

**Conclusions:**

PBSL has worse survival outcomes compared to tumors in other sites. HD-MTX chemotherapy remains effective, achieving a high ORR. Radiotherapy is an important salvage alternative for rapidly reducing brainstem lesions.

Implications for practice Most brain lesions in primary central nervous system lymphoma are supratentorial. Involvement of the brainstem is rare and generally has a poor prognosis. Currently, most studies consist of case reports. We retrospectively collected data on 56 patients with primary brainstem lymphoma (PBSL) to characterize clinical, therapeutic, and prognostic factors. Our study indicates that PBSL has worse survival outcomes compared to tumors in other sites. High-dose methotrexate-based chemotherapy achieves a high overall response rate in PBSL. For relapsed and refractory patients, radiotherapy may need to be fully considered for rapidly reducing brainstem lesions. The recent introduction of novel therapeutic agents may herald new hope for the treatment of PBSL.

## Introduction

Primary central nervous system lymphoma (PCNSL) is an aggressive and rare extranodal non-Hodgkin lymphoma limited to the brain, spine, cerebrospinal fluid (CSF), leptomeninges, and/or eyes.^1^ PCNSL accounts for 4% of primary brain tumors and 4%-6% of all extranodal lymphomas.^2^ The most common site of involvement is supratentorial rather than infratentorial; within the infratentorial region, the cerebellum is the most commonly involved site.^3^ Obtaining tissue from the brainstem for histopathological and molecular diagnosis presents significant challenges, complicating accurate diagnosis and subsequent treatment.^4^ Therefore, primary brainstem lymphoma (PBSL) is a rare and poorly investigated disease. Importantly, the involvement of deep brain or infratentorial structures, including the brainstem, periventricular regions, basal ganglia, and cerebellum in PCNSL, has recently been identified as an independent poor prognostic factor.^5^,6 In recent years, the introduction of novel targeted agents has offered additional treatment options for patients with poor clinical outcomes.^7^ However, there remains limited data regarding PBSL as a population with a poor prognosis.

Currently, the literature comprises only a few case reports (Table 1) and one study based on the Surveillance, Epidemiology, and End Results (SEER) database.^8^-^17^ Our study aimed to characterize clinical features, treatment methods, and identify prognostic factors associated with survival. We provide further information on this rare tumor entity and discuss the optimal treatment pattern.

**Table 1.** Recent case reports of primary brainstem lymphoma.

Reference	Gender and age	Signs and symptoms	Location	Number of brainstem lesions	Histopathology	Therapy	Overall survival
Kim^8^	9-year-old male	Diplopia, dizziness, dysarthria, right side hemiparesis	pons	Single	DLBCL	Radiosurgery and subsequent adjuvant chemotherapy (HD-MTX + vincristine + procarbazine)	10 mo
Jianhua^9^	30-year-old male	Rapid eye movement sleep behavior disorder	Pons + Midbrain	Single	DLBCL	HD-MTX + Ara-C	NA
Laigle-Donadey^10^	54-year-old male	Central neurogenic hyperventilation	Pons	Single	DLBCL	HD-MTX + lomustine + procarbazine	NA
Campbell^11^	55-year-old female	Dyskinesia, diplopia, headache	Pons	Single	DLBCL	Stereotactic radiosurgery	10 mo
Alsherbini^12^	49-year-old female	Ataxia, dysarthria, right hemiparesis, cognitive decline, headache	Pons + Midbrain	Single	DLBCL	HD-MTX + ritux-imab + temozolomide	NA
Shams^13^	54-year-old male	Binocular vertical diplopia, dysphagia, dyskinesia	Pons + Midbrain	Single	DLBCL	Supportive treatment	2 mo
Larner^14^	73-year-old male	Speech disturbance, unsteady gait, numbness of the right side of his face	Pons	Multiple	DLBCL	Supportive treatment	NA
Ficker^15^	68-year-old male	Autonomic dysfunction	Medulla	Single	DLBCL	NA	NA
McCue^16^	60-year-old male	Dysarthria, dysphagia, vertigo, nausea, horizontal diplopia, gait instability	Pons + Medulla	Multiple	T-cell lymphoma	Supportive treatment	2 mo

Abbreviations: Ara-C, cytarabine; DLBCL, diffuse large B-cell lymphoma; HD-MTX, high-dose methotrexate; mo, months.

## Methods

### Patient selection

The data of 56 patients diagnosed with PBSL at Beijing Tiantan Hospital between January 2018 and December 2022 were retrospectively analyzed.

The brainstem was subdivided into 3 segments: the superior (midbrain), middle (pons), and inferior parts (medulla). The tumor was defined as a brainstem lymphoma when more than 50% of the tumor involved the brainstem at diagnosis, ruling out primarily cerebellar or supratentorial tumors with extension to the brainstem.

**Figure 1.**
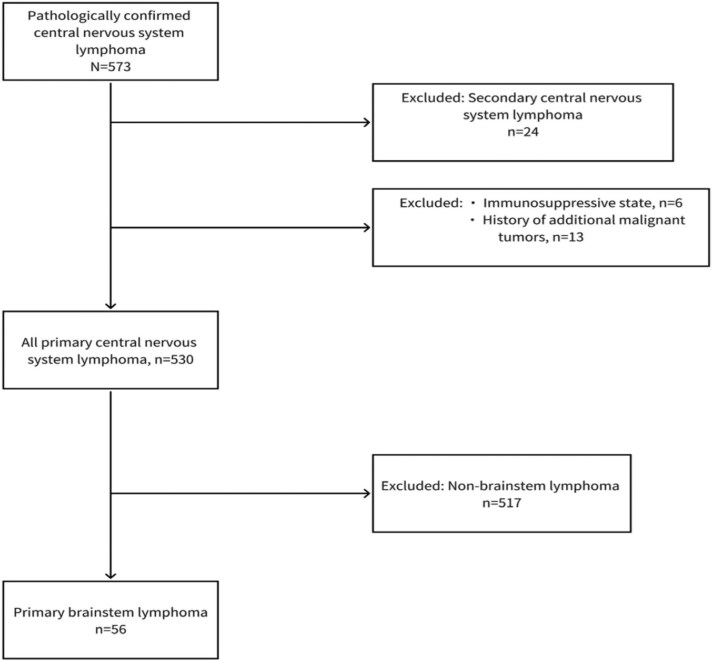
Flowchart of patient inclusion and exclusion.

The inclusion criteria were the following: (1) tissue samples were available through stereotactic biopsy or surgical resection; (2) completed cranial MRI, chest and abdominal CT or PET-CT, blood routine examination, and liver and kidney function tests were performed before treatment. The exclusion criteria were (1) immunosuppressive state; (2) additional malignant tumors discovered during the observation period; and (3) evidence of systemic involvement. Figure 1 shows the flowchart summarizing the patient selection process.

### Data collection

Data on the clinical course of disease, pathological parameters, neuroradiological imaging, laboratory results, and follow-up data were collected and analyzed from medical records. We evaluated the treatment responses as complete response (CR), partial response (PR), stable disease (SD), and progressive disease (PD) according to the International Workshop to Standardize Response Criteria for non-Hodgkin’s lymphoma.^18^ For all cases, the baseline and post-treatment MRI scans, including tumor location, size, and the presence of postcontrast enhancement, were reviewed alongside clinical records and follow-up data to evaluate treatment response. If imaging was not available, the evaluation was based on neuroradiology reports and clinic notes. Overall survival (OS) was calculated in months and defined as the period from the date of diagnosis to either the date of death for any cause or the last follow-up date; progression-free survival (PFS) was defined as the period from the date of diagnosis to the date of progression, relapse, or death.

### Statistical analysis

 OS and PFS were analyzed using the Kaplan–Meier method, and the differences between groups were compared using the log-rank test. The Cox proportional hazards regression model was used for both univariate and multivariate analyses. All tests were 2-tailed, and the significance level was set at *P* < .05. Data analyses were performed using SPSS (version 26.0; IBM Corp.) and R software (version 4.0.2).

## Results

### Patient characteristics

**Figure 2.**
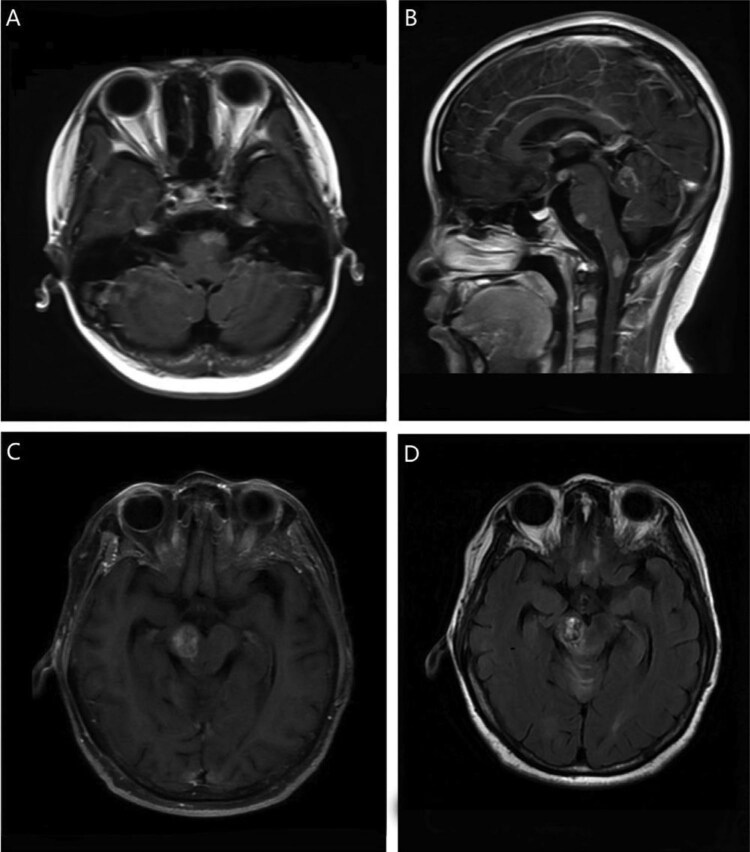
A 10-year-old female with primary brainstem lymphoma (PBSL). Axial (A) and sagittal (B) T1-postcontrast MR sequences showed multiple intense enhancing lesion in the medulla and pons. A 58-year-old female with PBSL. Axial T1-postcontrast MR sequence (C) showed an intense enhancing lesion in the midbrain. T2-FLAIR MR sequence (D) showed heterogeneous hyperintensity in the midbrain.

Patient characteristics are given in Table 2. The median age at diagnosis was 56 years (range: 10-77), with 64.3% of patients older than 60 years. Twenty-three patients (41.1%) had a Karnofsky Performance Score (KPS) ≤60. The most common symptoms were dyskinesia (61.4%), signs of increased intracranial pressure (47.3%), dizziness (45.2%), visual abnormality (34.5%), cognitive impairment (21.2%), and drowsiness (10.5%). Other rare symptoms included dysarthria and epilepsy (7.0%). Elevated LDH levels were observed in 12 patients (21.4%), and lymphopenia (≤1.0 × 109/L) was noted in 14 patients (25.0%). The midbrain was involved in 35.7% of patients, the pons in 23.2%, and the medulla in 12.5%. Involvement of 2 segments of the brainstem was present in 28.6% of cases, with infiltration of the midbrain and pons in 25.0% and of the pons and medulla in 3.6%. Fifty patients (89.3%) had a single brainstem lesion (Figure 2A and B), and 6 patients (10.7%) had multiple brainstem lesions (Figure 2C and D). Pathological tissue was obtained through stereotactic biopsy in 88% of cases (49 patients) and through surgical resection in 12.5% (7 patients). Histopathology was diffuse large B-cell lymphoma (DLBCL) in all patients. According to the Hans algorithm, 35 patients (62.5%) were classified as nongerminal center B cell (non-GCB), and 14 (23.2%) as germinal center B cell (GCB). Fourteen patients (25.0%) were positive for the double expressor of C-MYC and BCL-2, and 31 patients (55.4%) had a Ki-67 index exceeding 90%. Fifteen patients (26.8%) exhibited expression of CD3 + T.

**Table 2.** Patient characteristics.

Characteristics	N (%) or median (range)
Age	
Median (range)	56 (10-77)
<60	20 (35.7)
≥60	36 (64.3)
Gender	
Male	31 (55.4)
Female	25 (44.6)
KPS	
>60	33 (58.9)
≤60	23 (41.1)
LDH elevated	
Yes	14 (25.0)
No	39 (69.6)
NA	3 (5.4)
Lymphopenia	
Yes	14 (25.0)
No	39 (69.6)
NA	3 (5.4)
Location	
Midbrain	20 (35.7)
Pons	13 (23.2)
Medulla	7 (12.5)
Midbrain + pons	14 (25.0)
Pons + Medulla	2 (3.6)
Multiple brainstem lesion	
Yes	6 (10.7)
No	50 (89.3)
Diagnostic approaches	
Resection	7 (12.5)
Biopsy	49 (87.5)
Cell of origin	
Non-GCB	35 (62.5)
GCB	13 (23.2)
NA	8 (14.3)
Ki-67	
<90%	20 (35.7)
≥90%	31 (55.4)
NA	5 (8.9)
Double expressor (BCL-2 and C-MYC)	
Yes	14 (25.0)
No	26 (46.4)
NA	16 (28.6)
First-line chemotherapy	
Rituximab + HD-MTX	19 (33.9)
Rituximab + HD-MTX + Ara-C	20 (35.7)
MT-R	5 (8.9)
Other (eg, HD-MTX + IFO + Ara-C)	12 (21.5)
MSKCC	
Low	18 (32.1)
Intermediate	17 (30.4)
High	21 (37.5)

Abbreviations: Ara-C, cytarabine; IFO, ifosfamide; GCB, germinal center B cell; HD-MTX, high-dose methotrexate; KPS, Karnofsky Performance Score; LDH, lactate dehydrogenase; MSKCC, Memorial Sloan Kettering Cancer Center; MSKCC score: class 1 (patients <50 years), class 2 (patients ≥50; KPS ≥ 70) and class 3 (patients ≥50; KPS < 70).

**Table 3.** Cox’s regression analysis of OS and PFS.

Variable	Univariate analyses			Multivariate analyses		
	HR	95% CI	P	HR	95% CI	P
Overall survival						
Age ≥ 60	3.387	1.647-6.965	.001	3.086	1.467-6.492	.003
KPS ≤ 60	2.638	1.281-5.432	.008	1.524	0.609-3.809	.368
Elevated LDH	1.751	0.514-5.966	.371			
Location						
Midbrain			.752			
Pons	1.407	1.564-3.509	.464			
Medulla	1.277	0.406-4.022	.676			
Multiple brainstem lesion	2.672	1.056-6.761	.038	1.881	0.718-4.929	.199
Yes						
No						
Cell of origin (GCB versus Non-GCB)	1.119	0.469-2.672	.800			
Double expressor (BCL-2 and C-MYC)	1.081	0.418-2.794	.873			
Ki-67 ≥ 90%	1.177	0.553-2.504	.672			
First-line chemotherapy			.367			
Rituximab + HD-MTX + Ara-C	1.909	0.780-4.674	.157			
Rituximab + HD-MTX	1.499	0.183-12.259	.706			
MT-R						
Progression-free survival						
Age ≥ 60	2.321	1.237-4.358	.009	2.309	1.087-4.905	.030
KPS ≤ 60	2.542	1.321-4.892	.005	1.010	0.480-2.125	.979
Elevated LDH	1.934	0.772-4.843	.159			
Location						
Midbrain			.764			
Pons	1.216	0.509-2.903	.660			
Medulla	1.408	0.550-3.605	.476			
Multiple brainstem lesion	1.520	0.625-3.695	.356			
Yes						
No						
Cell of origin (GCB versus Non-GCB)	1.227	0.543-2.772	.622			
Double expressor (BCL-2 and C-MYC)	1.093	0.498-2.398	.824			
Ki-67 ≥ 90%	1.033	0.519-2.057	.926			
First-line chemotherapy			.428			
Rituximab + HD-MTX + Ara-C	2.183	0.279-17.077	.457			
Rituximab + HD-MTX	3.099	0.410-23.438	.273			
MT-R						

Abbreviations: Ara-C, cytarabine; IFO, ifosfamide; GCB, germinal center B cell; HD-MTX, high-dose methotrexate; KPS, Karnofsky Performance Score; LDH, lactate dehydrogenase; MSKCC, the Memorial Sloan Kettering Cancer Center; MSKCC score: class 1 (patients < 50 years), class 2 (patients ≥ 50; KPS ≥ 70) and class 3 (patients ≥ 50; KPS < 70).

### Treatment

No one received radiotherapy alone as an initial treatment. As part of their treatment, 78.5% of patients received rituximab. The most common first-line chemotherapy was rituximab + high-dose methotrexate (HD-MTX) in 33.9% of patients, rituximab + HD-MTX + cytarabine in 35.7% of patients, and rituximab + HD-MTX + temozolomide in 8.9% of patients. Other regimens included HD-MTX + ifosfamide + cytarabine and HD-MTX + ifosfamide + etoposide. The overall response rate (ORR) to first-line chemotherapy was 70.5%, with 62.1% of patients achieving a CR. SD was observed in 14.3%, PD in 12.7%, and response data were unavailable or unknown for 4.1%. Twenty-seven patients (48.2%) had a relapse or refractory disease. For these patients, various treatments were employed. Whole-brain radiation therapy (WBRT) was the most common treatment (*n* = 11). Other regimens included rituximab + HD-MTX + lenalidomide, rituximab + HD-MTX + cytarabine + etoposide + dexamethasone, ibrutinib + rituximab + HD-MTX + temozolomide, and a small portion of patients participated in clinical trials. Among patients receiving radiotherapy for relapsed or refractory lymphoma, 36.1% achieved CR, 27.8% achieved PR, and 70.6% were alive 1 year after radiotherapy.

### Survival outcomes and prognostic factors

**Figure 3.**
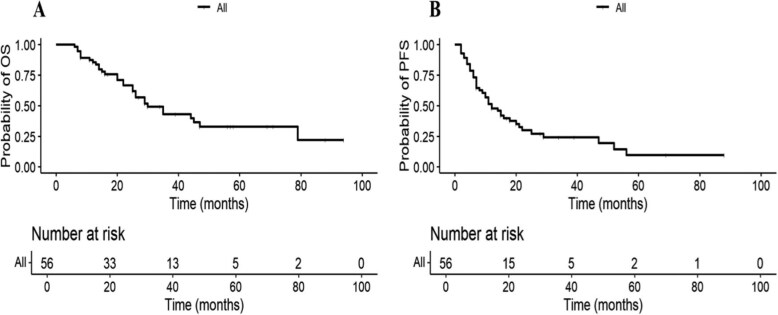
Kaplan–Meier curves for overall survival (OS) (A) and progression-free survival (PFS) (B) in patients with primary brainstem lymphoma (PBSL).

**Figure 4.**
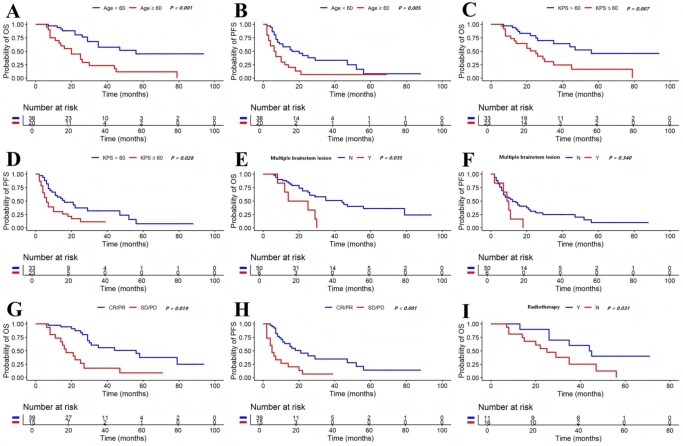
Kaplan–Meier curves for overall survival (OS) according to age (A), Karnofsky Performance Score (KPS) (C), number of brainstem lesions (E), and response to initial treatment (G). Kaplan–Meier curves for progression-free survival (PFS) according to age (B), KPS (D), number of brainstem lesions (F), and response to initial treatment (H). Kaplan–Meier curves for OS according to radiotherapy (I) in relapsed or refractory patients.

With a median follow-up of 56 months, 29 patients died, and the median OS was 30 months (95% CI 18-42 months, Figure 3A). The 3-year and 5-year OS rates were 44.7% and 28.6%, respectively. The median PFS was 11 months (95% CI 5-17 months, Figure 3B). The 3-year and 5-year PFS rates were 48.4% and 30.2%, respectively. In the univariate analysis (Table 3), age ≥60 years, KPS ≤60, and multiple brainstem lesions were significantly associated with poor OS. Age ≥60 years and KPS ≤60 were significantly associated with poor PFS. However, location, cell of origin, LDH level, BCL-2 and C-MYC co-expression, ki-67 index, or treatment regimens had no significant correlation with prognosis (*P* > .05). The Kaplan–Meier survival curves are shown in Figure 4A–F. In multivariate analysis (Table 3), age at diagnosis (age ≥60) was identified as a factor associated with poor OS (HR 3.086, *P* = .003, 95% CI 1.467-6.492) and PFS (HR 2.309, *P* = 0.030, 95% CI 1.087-4.905). Additionally, as shown in Figure 4G and H, the response to initial treatment significantly impacted long-term prognosis. Patients achieving CR or PR after first-line chemotherapy had a higher median OS (44 months, 95% CI 8-79 months) and PFS (21 months, 95% CI 10-31 months) compared to those experiencing SD or PD (median OS 22 months, 95% CI 11-32 months). The median OS for relapsed or refractory patients receiving radiotherapy was 44 months, compared to 25 months for those who did not receive radiotherapy (*P* = .031, Figure 4I).

## Discussion

The incidence of PBSL is very low. Among adult patients with brainstem lesions, lymphoma only accounts for approximately 10%.^19^ Most lesions in PCNSL are supratentorial (87%), frequently involving the frontal lobes (39%). When lesions occur infratentorially, the cerebellum is most commonly involved. Brainstem involvement has been described in approximately 3%-14% of newly diagnosed PCNSL.^20^-^22^ Our study represents the largest cohort of histopathologically confirmed PBSL cases to date. We collected data on 56 patients with PBSL to characterize clinical, therapeutic, and prognostic factors. Similar to supratentorial tumors, we observed PBSL mainly affected individuals over 60 years old, with a higher incidence in men than in women. Occurrence in children is extremely rare. In our cohort, 3 patients (3/56) were younger than 18 years old at the time of onset, with the youngest case being 10 years old, the youngest reported case of PCNSL in China. While it is well known that the pons is the most common site for brainstem gliomas,^23^ our study indicates that in PBSL, the midbrain is more commonly involved than the pons and medulla.

The diagnosis of brainstem lymphoma is more challenging compared to other types of lymphoma due to the brainstem’s role in regulating vital functions such as respiration and circulation, as well as transmitting sensory and motor signals between the spinal cord and the brain. Minor injuries to the brainstem can cause serious consequences.^23^ Therefore, a comprehensive evaluation of the benefits and risks associated with obtaining brainstem tissue is essential. Schumacher et al. found that biopsy is rarely necessary for brainstem disease when clinical history and symptoms, laboratory data, and imaging follow-up are considered together.^24^ Kickingerer et al. published a large meta-analysis of 1480 cases of stereo-tactic biopsy for brainstem tumors, and reported a 96.2% diagnostic success rate, a 7.8% overall morbidity rate, a 1.7% permanent morbidity rate, and a 0.9% fatality rate.^25^ A prospective study of 46 cases of brainstem biopsy indicated that, compared with MRI alone, stereotactic biopsy is a safe way to obtain a reliable tissue diagnosis, which is necessary for treatment decision.^19^ Given that pathology is the gold standard for diagnosing PCNSL, our study included only patients with histopathologically confirmed brainstem lymphoma, and all histological types were DLBCL.

Because PCNSL develops rapidly, especially in dangerous locations such as the brainstem, initial treatment should be initiated promptly upon confirmation of the diagnosis. HD-MTX can overcome the blood–brain barrier and achieve therapeutic levels in the CSF. HD-MTX-based chemotherapy (MTX dose of 3.5-8 g/m^2^) is the standard for induction therapy.^26^ Ferreri et al. demonstrated that adding cytarabine to HD-MTX improved ORR, PFS, and OS.^27^ The International Extranodal Lymphoma Study Group-32 (IELSG32) randomized trial showed a significant benefit in OS with the addition of rituximab and thiotepa to conventional methotrexate– cytarabine combination (MATRix regimen).^28^ Other common regimens combined with MTX include MT-R (methotrexate, temozolomide, rituximab), R-MVP (rituximab, methotrexate, procarbazine, vincristine), and MBVP (methotrexate, teniposide, carmustine, prednisone). However, the superiority of any of these regimens remains controversial, as they have not yet been evaluated in head-to-head trials.^29^ Initial therapy for our patients was HD-MTX-based chemotherapy, achieving an ORR similar to that of supratentorial tumors. Similar to previous studies, no significant survival differences were observed between these regimens.

Despite therapeutic progress with initial HD-MTX-based chemotherapy, 30% of patients may be refractory to HD-MTX.^30^ Moreover, approximately 50% of patients experience relapse after an initial response.^31^ There is currently no standard approach to treating patients with relapsed or refractory disease. The choice of salvage treatment should be based on data from prospective studies, considering prior treatment and response, patient age, performance status, and comorbidities. Optional treatments include rechallenge with MTX, temozolomide, rituximab, pemetrexed, topotecan, and multiple novel therapeutic agents.^7^ For chemosensitive patients with a good performance status who have not previously received autologous stem cell transplantation, this remains an option for managing relapse.^32^

PCNSL is sensitive to radiotherapy. For patients who have not received radiotherapy as part of initial treatment, WBRT remains an alternative salvage option for rapidly reducing intracerebral lesions and improving symptoms. In our cohort, WBRT accounted for most salvage treatment (40.7%) and achieved an ORR of 63.9%, providing outcomes similar to those seen with supratentorial tumors. Notably, among relapsed or refractory PBSL patients, those who received WBRT had significantly improved OS outcomes compared to those who did not (44 vs 25 months). Although several studies have focused on whether WBRT can improve prognosis, the results remain controversial. In a retrospective study involving 27 consecutive patients with initial HD-MTX failure, WBRT provided a high response rate (74%) and a median survival of 10.9 months.^33^ Similarly, a recent study on salvage WBRT for relapsed or refractory PCNSL identified a relatively high response rate (79%). The median survival from WBRT was 16 months, and 54% were alive 1 year after WBRT.^34^ Treatment-related neurotoxicity has been a significant concern in clinical practice. Due to concerns over delayed neurotoxicity, the benefits of WBRT need to be reevaluated.^35^ However, there is a lack of data on the correlation between radiotherapy efficacy and PBSL, a unique population with an increased risk of poor clinical outcomes. Given the aggressive nature of PBSL, especially in relapsed or refractory patients with brainstem involvement, the disease can deteriorate rapidly and become life-threatening. At the very least, our study suggests that the role of radiotherapy in treating brainstem lymphoma may need to be fully considered and should not be readily dismissed. Prospective trials with larger numbers of patients are needed to define the efficacy of WBRT.

More promisingly, for relapsed or refractory PCNSL, a number of novel therapeutic agents have demonstrated efficacy. The Bruton tyrosine kinase (BTK) inhibitor ibrutinib and the immunomodulatory drug (IMiD) lenalidomide are included in the National Comprehensive Cancer Network guidelines based on multiple studies. Checkpoint inhibitors (pembrolizumab and nivolumab) and CD19-directed chimeric antigen receptor T-cell therapies have entered phase I and II clinical trials.^36^ Recent studies are contributing to our increasing understanding of PCNSL molecular heterogeneity and biological biomarker, providing a basis for subtype-based targeted interventions. Cooper et al. showed that CD5 IHC + DLBCLs had an enrichment for oncogenic BCR mutations known to correlate with sensitivity to BTK inhibitors.^37^ In our cohort, CD5 expression was observed in three patients (3/29), all of which were non-GCB DLBCLs. As these novel agents transition from clinical trials into routine clinical practice, we anticipate that they will offer additional treatment options for patients with PBSL.

Previous studies have shown that patients with brainstem lymphoma have a poor prognosis. The median survival time in our study was 30 months, which is shorter than the 33-57 months reported in recent series on PCNSL.^38^-^40^ The median PFS of 11 months was also shorter than that reported previously for PCNSL (13-17 months).^21^,41 The International Extranodal Lymphoma Study Group (IELSG) indicated that involvement of deep brain regions was significantly and independently associated with worse survival. In this 5-point scoring system based on age, Eastern Cooperative Oncology Group performance status (ECOG PS), serum LDH level, CSF total protein concentration, and involvement of deep brain regions, the brainstem is defined as one of the deep brain structures.^5^ A retrospective study involving 85 patients in the ANOCEF-GOELAS prospective randomized trial provided evidence that infratentorial location was associated with poor OS and PFS.^6^ Based on integrating genome-wide data from multi-omic data, Hernández-Verdin et al. identified 4 molecular patterns of PCNSL, which displayed different clinical outcomes in OS. Interestingly, CS2/CS3, which had a poorer OS than CS1/CS4, were more frequent in the brainstem. The heterogeneous-immune CS3 group had the worst prognosis, probably due to its association with enriched HIST1H1E mutations.^42^ Therefore, the poor prognosis in brainstem regions may be related to underlying molecular heterogeneity in PCNSL.

Age and PS are the only two universally accepted factors affecting the prognosis of PCNSL. We attempted to analyze the prognostic factors of our cohort and confirmed the strong prognostic impact of age, KPS, and the number of brainstem lesions. In the multivariate Cox regression analysis, KPS was not independently associated with prognosis. However, this may be attributed to the strong interaction between KPS and age.

This study has some limitations. It was a small and single-center study, including only 56 patients. Our data were collected retrospectively, which suffered from inevitable selection bias, variability in data collection, and a lack of standardized follow-up schedules. The median follow-up time was not sufficiently long, which affected the accuracy of the prognosis analysis.

## Conclusion

Stereotactic biopsy remains the procedure of choice to obtain a histological diagnosis for PBSL. Brainstem lymphoma has a poor prognosis and age (≥60) has a negative impact on OS and PFS. HD-MTX-based chemotherapy is the standard for induction therapy. The optimal salvage treatment regimen remains undefined. Currently, WBRT remains an important salvage option, though its efficacy needs validation in larger prospective trials. The remarkable progress in researching new drugs for lymphoma treatment, such as BTK inhibitors, immunocheckpoint inhibitors, XOP-1 inhibitors, may herald new hope for the treatment of PBSL.

## Data Availability

The data underlying this article will be shared on reasonable request to the corresponding author.
